# Multiple Myeloma of the Thyroid Cartilage

**DOI:** 10.1155/2012/194797

**Published:** 2012-04-29

**Authors:** Peter Kalina, Jeffrey B. Rykken

**Affiliations:** Department of Radiology, Mayo Clinic, Rochester, MN 55905, USA

## Abstract

A 60-year-old male presented with hoarseness. His past medical history was remarkable for a plasmacytoma of the left maxillary sinus having been resected without systemic evidence of plasma cell myeloma (PCM), also known as multiple myeloma (MM), at the time. This maxillary sinus disease recurred and was treated with radiation. Workup for PCM was conducted. Treatment included melphalan and autologous stem cell transplant. Because of the therapeutic and prognostic implications, a Plasma cell neoplasm (PCN) in a neck mass must be carefully evaluated by clinical and pathological criteria in order to distinguish plasmacytoma from PCM. PCN involvement of the thyroid cartilage is very rare, with only 5 previously reported cases.

## 1. Introduction

Plasma cell neoplasms (PCNs) include extramedullary plasmacytoma (EMP), solitary bone plasmacytoma (SBP), and manifestation of plasma cell myeloma (PCM). EMP and SBP are benign and local. PCM is a systemic process and the most common PCN with the worst prognosis. Workup for PCM generally includes serum electrophoresis with evaluation for the presence of a monoclonal peak, consisting of IgG with kappa light chain. This case demonstrates the possibility of an indolent course of PCM with destructive laryngeal plasmacytoma without systemic findings. PCN accounts for less than 1% of head/neck tumors. Furthermore, PCN of the thyroid cartilage is very rare.

## 2. Case Report

**Figure 1:**
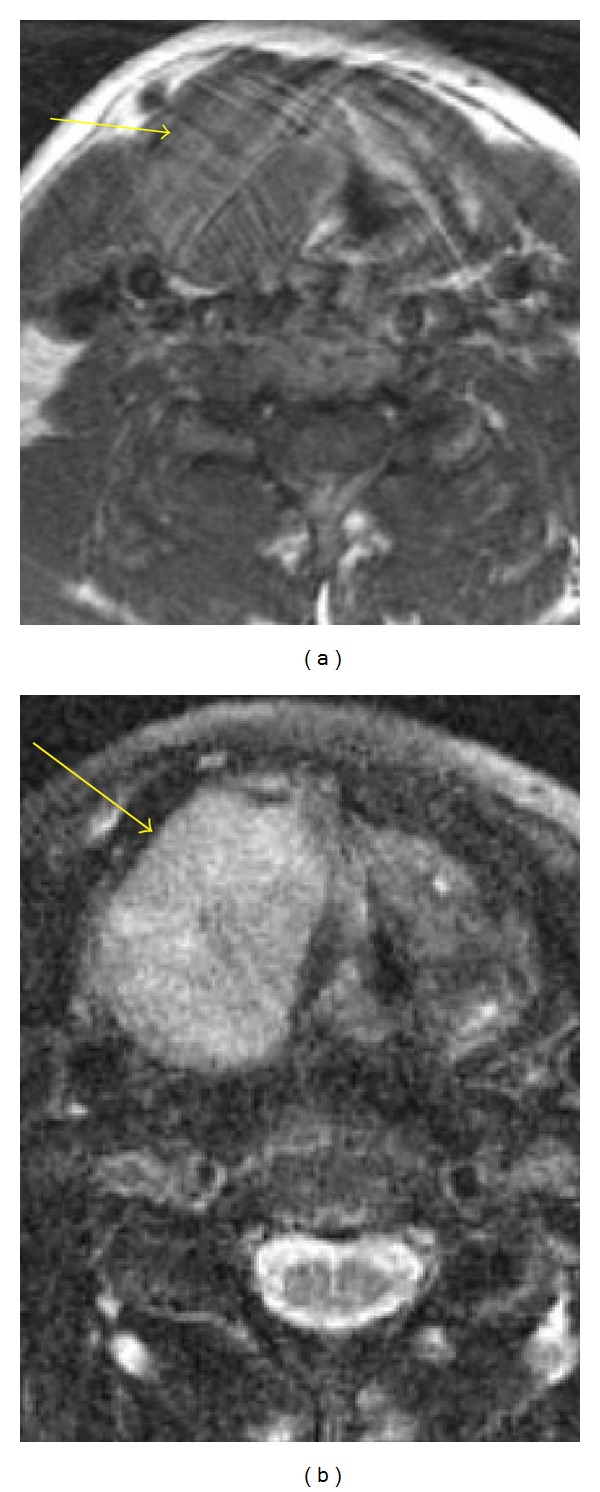
Axial T1 (a) and T2 (b), noncontrast: homogeneous, well-defined mass of the right thyroid cartilage laminae.

**Figure 2:**
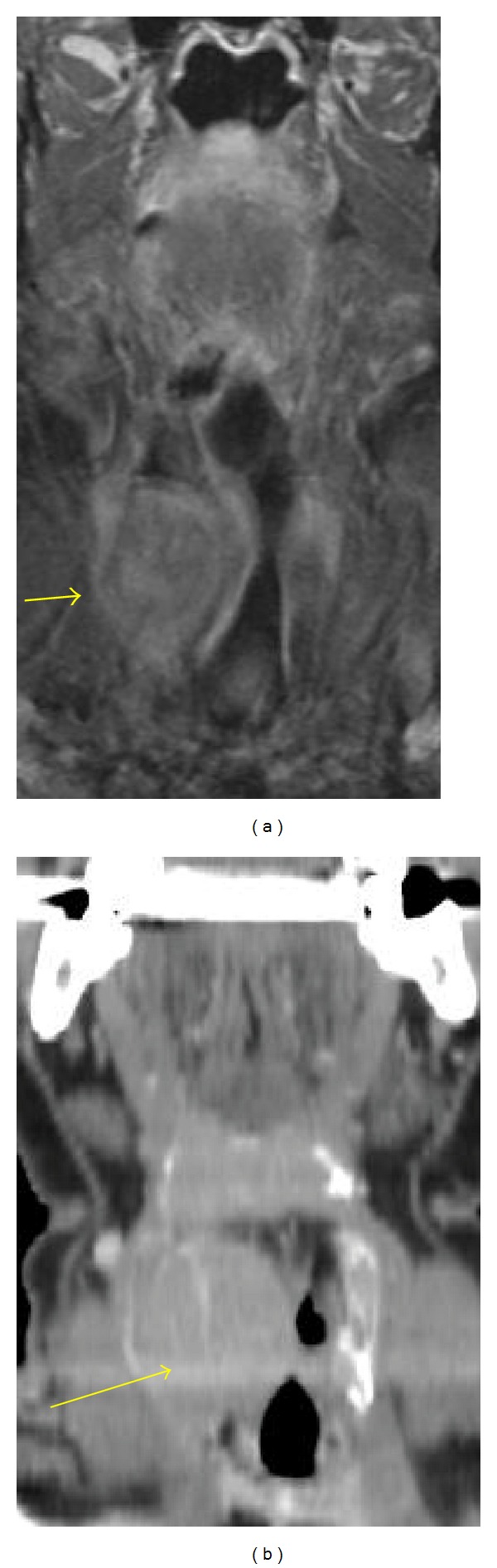
Coronal MRI (a) and coronal CT (b); postcontrast homogeneous enhancement of the mass.

**Figure 3:**
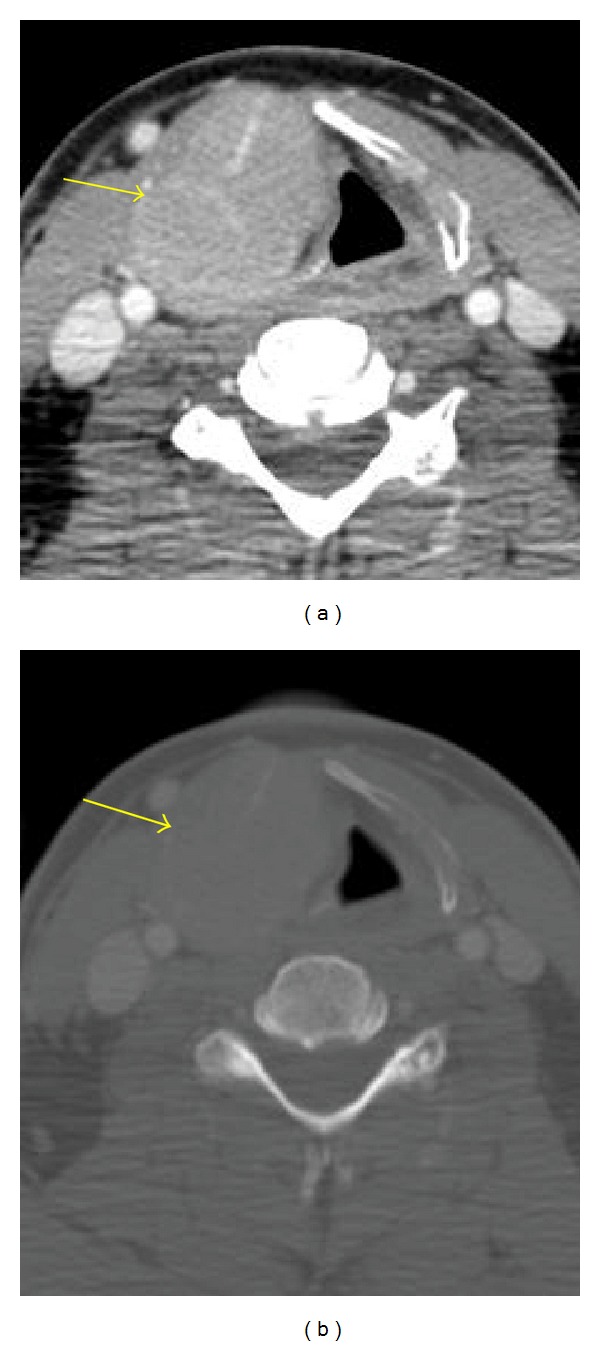
CT, postcontrast, soft tissue (a) and bone windows (b) Uniformly expanded right thyroid cartilage laminae.

**Figure 4:**
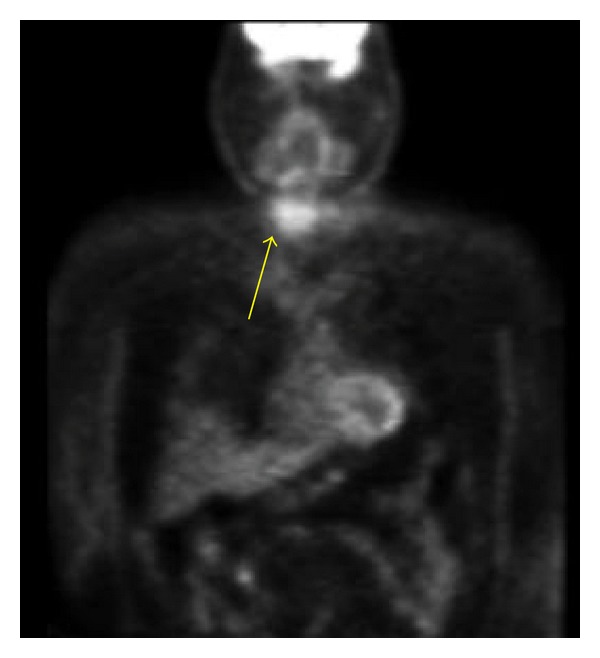
Nuclear Medicine Bone Scan: Confirms increased uptake of the lesion.

A 60-year-old male presented with a three-month history of increasing hoarseness. Imaging was obtained consisting of a soft tissue neck MRI with noncontrast axial sequences (Figure 1), postcontrast coronal MRI and CT (Figure 2), postcontrast axial CT (Figure 3) and a nuclear medicine bone scan (Figure 4). His past medical history was remarkable for a plasmacytoma of the left maxillary sinus having been resected, although there was no systemic evidence of PCM at the time. This maxillary sinus disease recurred and was treated with radiation. Workup for PCM included serum electrophoresis demonstrating a monoclonal peak consisting of IgG with kappa light chain.

Initial bone marrow analysis demonstrated a slight increase in erythroid precursors and a slight decrease in granulocytic precursors. Plasma cells were borderline increased and estimated at 2-3% of the total cellularity. Immunohistochemical studies were essentially normal, other than a slight increase in plasma cell population identified via staining for CD138.

An additional bone marrow analysis was performed nearly seven months later. At this time, the patient was five-years out from the initial plasmacytoma resection. On this second marrow evaluation, panmyeloid hyperplasia was present and a tiny aggregate of atypical plasma cells was identified and deemed worrisome for early involvement of plasma cell neoplasm. Immunophenotyping was unable to be performed as too few plasma cells were present.

He initially had an early stage, indolent course of PCM with a destructive laryngeal plasmacytoma without systemic findings. In fact, an advanced destructive laryngeal lesion may exist with indolent PCM. Later skeletal surveys demonstrated multiple lytic lesions throughout the axial and appendicular skeleton. Treatment included high-dose melphalan and autologous stem cell transplant.

## 3. Discussion

Plasma cell neoplasms (PCN) represent a monoclonal plasma cell proliferation. The three types, extramedullary plasmacytoma (EMP), solitary bone plasmacytoma (SBP), and manifestation of PCM (or multiple myeloma), represent distinct manifestations of the same disease, representing a disease continuum [1]. EMP (3% of PCN) is a local plasma cell tumor outside of marrow. Of these, 50–60% progress to PCM [2]. The larynx is involved in 5–20% cases but rarely involves the cartilage. SBP represents a local marrow plasma cell proliferation manifesting as an intraosseous lytic lesion. Of these, 20–30% progress to PCM [2]. EMP and SBP are benign and local, without systemic disease or criteria of MM based on negative electrophoresis, bone marrow biopsy, and radiographs. PCM is the most common PCN. It is a systemic disease with disseminated involvement and thus has the worst prognosis. Findings include bone pain, anemia, renal failure, marrow plasmacytomas, lytic bone lesions, and a monoclonal gamma globulin peak. Patients may also have clinically silent disseminated extraosseous disease.

PCN accounts for less than 1% of head/neck tumors [6]. EMP is usually a submucosal soft tissue mass in a paranasal sinus, nasal cavity, or nasopharynx. Most PCNs of the larynx represent EMP without PCM rather than extraosseous PCM. The most common symptom of laryngeal plasmacytoma is slowly progressive hoarseness. PCN of the thyroid cartilage is very rare.

There are two theories to explain cartilage involvement [3]: direct cartilage invasion by adjacent plasmacytoma or metaplasia of cartilage to bone with formation of a marrow cavity in which a plasmacytoma originates. It is considered an extraosseous manifestation of PCM when cartilage is involved because it occurs in ossified cartilage rather than bone. It is rare compared with intramedullary PCM. Lesions are typically homogeneous, well defined, and enhancing. Uniformly expanded thyroid cartilage laminae and no soft tissue mass suggest that a plasmacytoma originates in the thyroid ala rather than being eroded by adjacent soft tissue plasmacytoma.

The three PCN variants are histologically indistinguishable. Because of the therapeutic and prognostic implications, a PCN in a neck mass must be carefully evaluated by clinical and pathological criteria in order to distinguish plasmacytoma from PCM.

Treatment of PCN of the larynx is local or wide excision as well as radiation (EMP/SBP are radiosensitive). Treatment of PCM includes melphalan/prednisolone, radiation for local control, and autologous bone marrow transplant with peripheral blood stem cells.

PCN involvement of the thyroid cartilage is very rare, with only 6 previously reported cases (Table 1) [1–6]. In 3 of these cases, the neck mass was the initial presenting feature of PCN. In 4 of 7 cases, including this case, hoarseness was a presenting feature of the thyroid cartilage lesion.

## Figures and Tables

**Table 1:** Comparison of published reports of plasma cell myeloma (or multiple myeloma) with thyroid cartilage involvement.

Authors	Demographics	Initial diagnosis of multiple myeloma	Presentation of thyroid cartilage lesion	Imaging	Skeletal survey at time of presenting thyroid lesion	Treatment of thyroid lesion
Van Dyke C et al. [3]	62-year-old male	Expansile lytic rib lesion	Worsening hoarseness for 6 months and neck swelling for weeks	Contrast-enhanced CT	Multiple bony lesions on radiographic survey	Radiation therapy
Saad R et al. [4]	79-year-old male	Osteolytic lesion of 6th thoracic vertebra	Neck mass associated with pain upon swallowing	Contrast-enhanced CT	Multiple lytic bony lesions on radiographic survey	Not reported
Gross M et al. [5]	50-year-old male	Neck mass	Progressively enlarging neck mass for 6 months with respiratory distress and stridor	Non-contrast CT	Multiple lytic bony lesions on radiographic survey	Radiation and chemotherapy (vincristine, doxorubicin, and dexamethasone; later changed to melphalan)
Aslan I et al. [1]	70-year-old male	Neck mass	Slight hoarseness and neck fullness for 4 months	Non-contrast CT	No lesions demonstrated	Debulking of mass and radiation therapy
Sosna J et al. [6]	54-year-old male	Neck mass	Recent neck lump detected by patient with increasing hoarseness and progressive dyspnea	Non-contrast CT	Multiple lytic bone lesions on radiographic survey	Radiation and chemotherapy (vincristine, doxorubicin, and dexamethasone; later given melphalan)
Shimada T et al. [2]	72-year-old male	Pathologic cervical vertebral body fracture	Asymptomatic, incidentally discovered on CT of cervical spine	Non-contrast CT	No additional lesions on whole body scintigraphy	Chemotherapy and/or bone marrow transplant felt indicated, but no therapy given, due to patient's age and dementia.
Kalina P et al. [7]	60-year-old male	Nasolacrimal duct obstruction with epiphoria due to paranasal sinus mass	Hoarseness for 3 months	Contrast-enhanced MRI; contrast-enhanced CT, and bone scan	Two lytic lesions, one each of the skull and left humerus	Autologous bone marrow transplant
